# Development and validation of a nomogram to predict overall survival in young non-metastatic rectal cancer patients after curative resection: a population-based analysis

**DOI:** 10.1007/s00384-022-04263-y

**Published:** 2022-10-21

**Authors:** Zhenya Jia, Huo Wu, Jing Xu, Guoping Sun

**Affiliations:** 1 Department of Medical Oncology, The First Affiliated Hospital of Anhui Medical University, 218 Jixi Road, Hefei, 230022 People’s Republic of China; 2 Department of General Surgery, The First Affiliated Hospital of Anhui Medical University, Hefei, 230022 People’s Republic of China

**Keywords:** Rectal cancer, Nomogram, Young patients, Overall survival

## Abstract

**Purpose:**

This study aimed to establish and validate a nomogram for predicting overall survival (OS) in young non-metastatic rectal cancer (RC) patients after curative resection.

**Methods:**

Young RC patients (under 50 years of age) from 2010 to 2015 were extracted from the Surveillance, Epidemiology, and End Results (SEER) database. Those patients randomly assigned to a training cohort and a validation cohort at a ratio of 7:3. The independent prognostic factors for OS were identified by univariate and multivariate Cox regression analysis. A nomogram model was built based on the independent prognostic variables and was evaluated by concordance index (C-index), receiver operating characteristics (ROC) curves, calibration plot, and decision curve analysis (DCA).

**Results:**

A total number of 3026 young RC patients were extracted from SEER database. OS nomogram was constructed based on race, histological type, tumor grade, T stage, N stage, carcinoembryonic antigen (CEA) level, and number of lymph nodes (LN) examined. C-index, ROC curves, calibration plot, and DCA curves presented satisfactory performance of the above nomogram in predicting the prognosis of young non-metastatic RC patients after curative resection. The nomogram can identify three subgroups of patients at different risks, which showed different prognostic outcomes both in the training cohort and validation cohort.

**Conclusion:**

We successfully established a reliable and insightful nomogram to predict OS for young non-metastatic RC patients after curative resection. The nomogram may provide accurate prognosis prediction to guide individualized follow-up and treatment plans.

## Introduction

Rectal cancer (RC) represents the eighth most frequently diagnosed cancer worldwide, causing about 339 thousand deaths in 2020 [1]. RC is traditionally known as a malignancy in the elderly. The incidence of patients with RC has shown a downward trend over the past few decades [2, 3]. Unfortunately, recent studies have reported an increasing incidence of young RC patients under 50 years of age [4–6]. RC patients at a young age tend to present with a more advanced tumor stage and worse biological behavior compared with elderly patients, which may be related with poor prognosis [7, 8]. Therefore, RC in young patients has attracted wide attention throughout the world.

For RC patients with stages I–III, curative resection is the primary treatment without controversy. Although neoadjuvant chemoradiotherapy and adjuvant chemoradiotherapy have been widely used in treating the disease and obtained positive effects, the prognosis of young non-metastatic RC patients is still poor. The tumor-node-metastasis (TNM) staging system which was developed jointly by the UICC (Union Internationale Against cancer) and the AJCC (American joint Committee on cancer) remains the gold standard to predict surgical outcomes for patients with RC. However, the prognosis was obviously different in young non-metastatic RC patients with the same stage after surgical resection, suggesting that the TNM staging system failed to provide individualized predictions. Thus, developing an accurate prediction model is necessary to effectively identify individuals with different survival risks, which may help guide clinical decision-making.

Nomograms have been regarded as reliable and effective tools to evaluate cancer outcomes by incorporating various pathological and clinical characteristics. It has been recognized that nomograms do better than traditional TNM staging systems in assessing the prognosis of cancer [9–11]. Nevertheless, there is no nomogram to predict the overall survival (OS) in young non-metastatic RC patients after curative resection.

Under this background, we aimed to develop a nomogram among young non-metastatic RC patients undergoing curative resection based on cases from Surveillance, Epidemiology, and End Results (SEER) database and validate its predictive accuracy.

## Materials and methods

### Database and variables

**Fig. 1:**
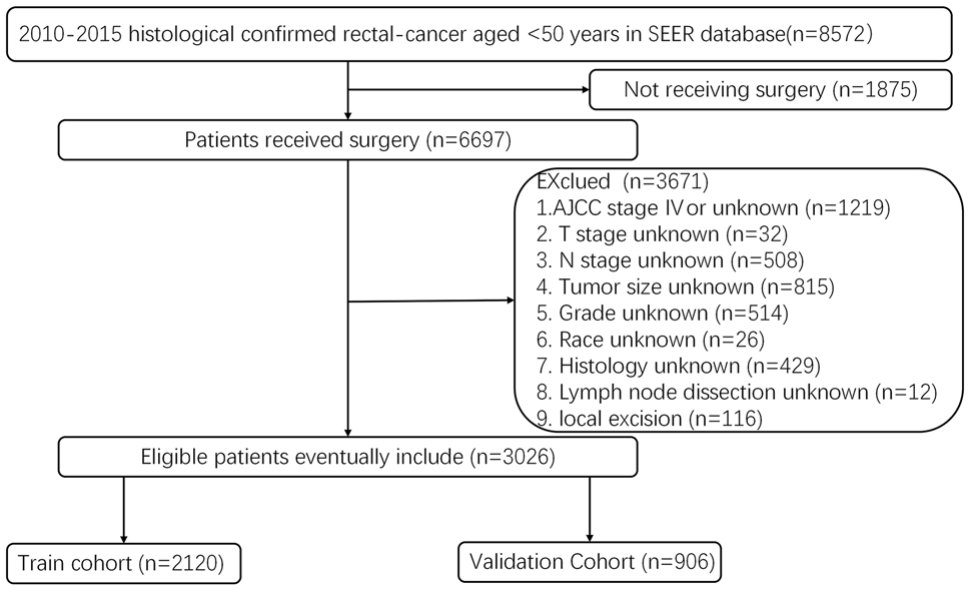
The flow diagram of the selection process

As an authoritative source for cancer statistics, the SEER database covers the cancer incidence data about approximately 47.9 percent of the US population. The SEER*Stat software (version 8.3.9) was used to retrieve the data of young RC patients (under 50 years of age) diagnosed between 2010 and 2015. The International Classification of Diseases for Oncology 3rd edition (ICD-O-3) was used to identify the diagnosis of RC. The 7th edition AJCC staging system was applied to estimate the stage of diseases. Since T stage and N stage can indirectly judge the overall TNM stage of non-metastatic RC patients and study the impact of primary tumor and lymph node status on prognosis respectively, this study mainly discussed T stage and N stage rather than overall TNM stage. OS was chosen as the primary endpoint of the study. OS was defined as the time from date of diagnosis of RC to the date of death from any cause or the last date of follow-up. The selection process is illustrated in Fig. 1.

The inclusion and exclusion criteria of this study were as follows.

The inclusion criteria: (1) patients with age under 50 years; (2) patients with resection of primary malignancies; (3) patients with histologically confirmed rectal cancer.

The exclusion criteria: (1) patients with more than one malignancy; (2) patients with survival time less than 1 month; (3) patients in AJCC stage IV or unknown; (4) patients with local excision; (5) patients with incomplete information of T stage, N stage, tumor size, grade, race, histology, and number of lymph nodes (LN) examined.

The variables included various clinicopathological characteristics such as age, sex, race, histological type, tumor grade, T stage, N stage, tumor size, carcinoembryonic antigen (CEA) level, number of lymph nodes (LN) examined, radiotherapy, chemotherapy, and year of diagnosis. Age was classified into < 20, 20 − 29, 30 − 39, and 40 − 49. Sex was classified into male and female. Race was classified into White, Black, and others. Histological type was classified into adenocarcinoma (ICD-O-3, 8140/3–8147/3, 8210/3–8213/3, 8255/3, 8260/3–8263/3), mucinous adenocarcinoma (ICD-O-3, 8480/3, 8481/3), and signet ring cell carcinoma (ICD-O-3, 8490/3). Tumor grade was classified into grade I, grade II, grade III, and grade IV. T stage was classified into T1, T2, T3, T4a, and T4b. N stage was classified into N0, N1a, N1b, N1c, N2a, and N2b. Tumor size was classified into < 5 cm and ≥ 5 cm. CEA level was classified into normal level, elevated level, and unknown. Number of LN examined was classified into < 12 and ≥ 12. Radiotherapy and chemotherapy were classified into receiving and not receiving. Year of diagnosis was classified into 2010–2012 and 2013–2015.

### Statistical analysis

All patients were randomly assigned to a training cohort and a validation cohort at a ratio of 7:3. All of the relevant variables were identified by univariate analysis. The potential prognostic variables with significant association (*p* < 0.05) to OS were analyzed using the multivariate Cox regression model to determine the independent prognostic factors. Based on the multivariate analysis results, the R software was used to construct an OS nomogram to predict the 3-, 5-, and 8-year survival rates in young non-metastatic RC patients after curative resection. C-index was used to evaluate the prediction accuracy of the nomogram. The receiver operating characteristics (ROC) curve was applied to assess the sensitivity and specificity. The calibration plot was used to evaluate the congruence between predicted and observed outcomes. Furthermore, decision curve analysis (DCA) was performed to estimate the clinical value of the nomogram. Kaplan–Meier survival analysis was delineated to evaluate the prognostic value of the nomogram. All statistical analyses were performed using R software (version 4.1.2). The X-tile software (version 3.6.1) was used to determine the optimal cutoff values for the nomogram. A two-tailed value of *p* < 0.05 was regarded as the statistically significant difference.

## Results

### Patient characteristics

According to the inclusion and exclusion criteria, a total of 3026 young RC patients were finally included in the analysis, of which 2120 patients were randomly assigned to the training cohort and the other 906 patients to the validation cohort. There was no significant difference between the training cohort and the validation cohort. In the training cohort, a majority of patients were in the age of 40 to 49 years (77.4%), male (54.8%), and White (79.2%). The most common histological type was adenocarcinoma (94.6%), followed by mucinous adenocarcinoma (4.7%), and signet ring cell carcinoma (0.7%). The most common tumor grade was grade II (79.6%), followed by grade III (11.4%), grade I (6.7%), and grade IV (2.3%). Most patients were diagnosed with T3 (61.4%), N0 (49.2%), and normal CEA level (42.5%). For the tumor size, more than half of the patients were < 5 cm (60.8%). Most patients received a number of LN examined ≥ 12 (82.1%), chemotherapy (74.6%), and radiotherapy (58.2%). The demographic and clinical characteristics of all the patients are exhibited in Table 1.

**Table 1:** Demographics and clinicopathological characteristics of 3026 patients

Variables	Training cohort		Validation cohort	
	*n* = 2120		*n* = 906	
	*n*	%	*n*	%
Age				
< 20	2	0.1	2	0.2
20–29	74	3.5	28	3.1
30–39	402	19.0	209	23.1
40–49	1642	77.4	667	73.6
Sex				
Male	1162	54.8	489	54.0
Female	958	45.2	417	46.0
Race				
White	1680	79.2	721	79.6
Black	175	8.3	77	8.5
Others	265	12.5	108	11.9
Histological type				
Adenocarcinoma	2006	94.6	862	95.1
Mucinous adenocarcinoma	99	4.7	36	4.0
Signet ring cell carcinoma	15	0.7	8	0.9
Tumor grade				
Grade I	143	6.7	68	7.5
Grade II	1687	79.6	693	76.5
Grade III	241	11.4	123	13.6
Grade IV	49	2.3	22	2.4
T stage				
T1	220	10.4	112	12.4
T2	397	18.7	156	17.2
T3	1301	61.4	554	61.1
T4a	107	5.0	41	4.5
T4b	95	4.5	43	4.8
N stage				
N0	1044	49.2	441	48.7
N1a	316	14.9	136	15.0
N1b	290	13.7	136	15.0
N1c	32	1.5	19	2.1
N2a	222	10.5	84	9.3
N2b	216	10.2	90	9.9
Tumor size				
< 5 cm	1288	60.8	547	60.4
≥ 5 cm	832	39.2	359	39.6
CEA				
Normal level	901	42.5	383	42.3
Elevated level	491	23.2	216	23.8
Unknown	728	34.3	307	33.9
Number of LN examined				
< 12	379	17.9	150	16.6
≥ 12	1741	82.1	756	83.4
Chemotherapy				
Receiving	1582	74.6	672	74.2
Not receiving	538	25.4	234	25.8
Radiotherapy				
Receiving	1233	58.2	508	56.1
Not receiving	887	41.8	398	43.9
Year of diagnosis				
2010–2012	1030	48.6	443	48.9
2013–2015	1090	51.4	463	51.1

*CEA* carcinoembryonic antigen, *LN* lymph nodes

### Factors associated with OS

**Fig. 2:**
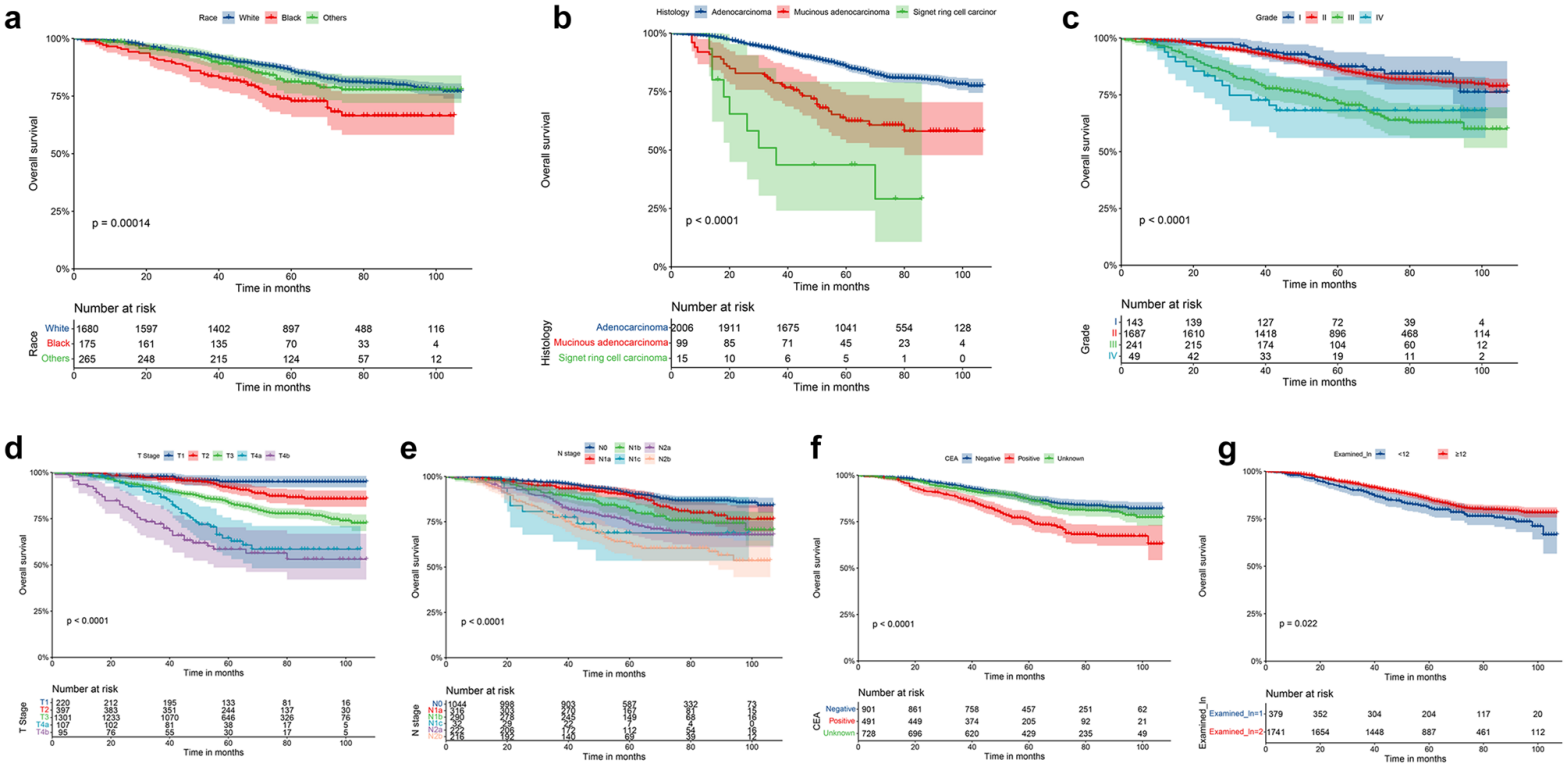
Kaplan–Meier survival curves analysis for OS stratified by **a** race, **b** histological type, **c** tumor grade, **d** T stage, **e** N stage, **f** CEA level in the training cohort, **g** number of LN examined. OS: overall survival; T, tumor; N, node; CEA, carcinoembryonic antigen; LN, lymph nodes

For the training cohort, sex, race, histological type, tumor grade, T stage, N stage, tumor size, CEA level, number of LN examined, radiotherapy, and chemotherapy were correlated with OS in the univariate analysis and subjected to the multivariate analysis. As shown in Table 2, race, histological type, tumor grade, T stage, N stage, CEA level, and number of LN examined were confirmed to be the independent prognostic factors for the OS of young non-metastatic RC patients after curative resection (*p* < 0.05). The associations between the independent prognostic factors (race, pathological type, tumor grade, T stage, N stage, CEA level, and number of LN examined) and OS are presented in Fig. 2.

**Table 2:** Univariate and multivariate analysis of OS in the training cohort

	Univariate analysis				Multivariate analysis			
Variables	HR	95%CI		*p* value	HR	95%CI		*p* value
Age								
< 20	1							
20–29	-	-	-	0.991	-	-	-	-
30–39	-	-	-	0.990	-	-	-	-
40–49	-	-	-	0.991	-	-	-	-
Sex								
Male	1							
Female	0.79	0.64	0.98	0.028	0.84	0.68	1.04	0.110
Race								
White	1							
Black	1.93	1.41	2.64	< 0.001	1.58	1.14	2.17	0.005
Others	1.2	0.88	1.64	0.244	1.11	0.81	1.52	0.511
Histological type								
Adenocarcinoma	1							
Mucinous adenocarcinoma	2.65	1.88	3.75	< 0.001	1.72	1.21	2.46	0.003
Signet ring cell carcinoma	6.5	3.35	12.63	< 0.001	3	1.48	6.1	0.002
Tumor grade								
Grade I	1							
Grade II	1.12	0.7	1.78	0.636	1.1	0.69	1.77	0.690
Grade III	2.66	1.6	4.4	< 0.001	1.76	1.05	2.95	0.032
Grade IV	2.93	1.49	5.76	< 0.001	2.05	1.03	4.08	0.042
T stage								
T1	1							
T2	2.23	1.11	4.45	0.024	1.91	0.95	3.85	0.069
T3	4.44	2.36	8.35	< 0.001	2.75	1.41	5,34	0.003
T4a	9.16	4.54	18.52	< 0.001	5.02	2.4	10.51	< 0.001
T4b	12.75	6.34	25.64	< 0.001	6.23	2.95	13.19	< 0.001
N stage								
N0	1							
N1a	1.45	1.03	2.06	0.035	1.35	0.94	1.93	0.101
N1b	2.11	1.53	2.89	< 0.001	1.75	1.26	2.43	< 0.001
N1c	3.84	1.94	7.58	< 0.001	2.61	1.3	5.23	0.007
N2a	2.89	2.11	3.97	< 0.001	2.24	1.61	3.11	< 0.001
N2b	4.54	3.38	6.1	< 0.001	3.17	2.29	4.39	< 0.001
Tumor size								
< 5 cm	1							
≥ 5 cm	1.64	1.33	2.02	< 0.001	1.13	0.9	1.41	0.283
CEA								
Normal level	1							
Elevated level	2.13	1.65	2.74	< 0.001	1.58	1.22	2.05	< 0.001
Unknown	1.16	0.9	1.5	0.253	1.19	0.91	1.54	0.198
Number of LN examined								
< 12	1							
≥ 12	0.75	0.58	0.96	0.022	0.59	0.46	0.77	< 0.001
Chemotherapy								
Receiving	1							
Not receiving	0.52	0.39	0.69	< 0.001	1.26	0.87	1.81	0.217
Radiotherapy								
Receiving	1							
Not receiving	0.63	0.51	0.79	< 0.001	0.79	0.6	1.04	0.094
Year of diagnosis								
2010–2012	1							
2013–2015	0.83	0.66	1.04	0.107	-	-	-	-

*OS* overall survival, *HR* hazard ratio, *CI* confidence interval, *CEA* carcinoembryonic antigen, *LN* lymph nodes

### Nomogram construction for young RC patients

**Fig. 3:**
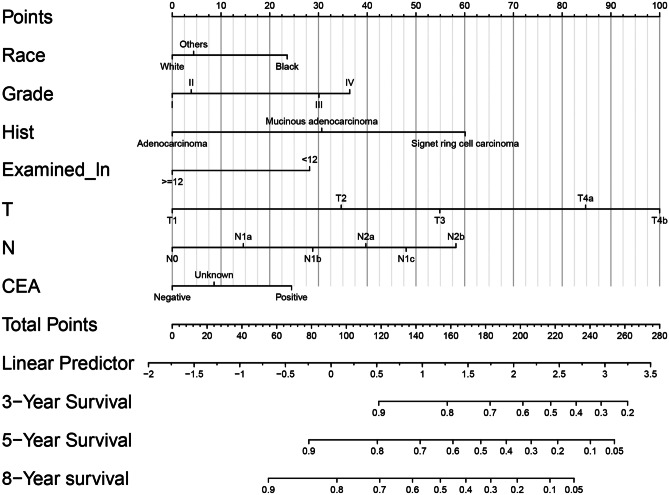
A nomogram to predict 3-, 5-, and 8-year OS for young non-metastatic RC patients after curative resection. OS: overall survival; RC; rectal cancer

All the above independent prognostic factors were used to create an OS prognostic nomogram. The nomogram for 3-, 5-, and 8-year OS is shown in Fig. 3. By adding up the scores to the bottom scales, we can predict 3-, 5-, and 8-year OS of individual young non-metastatic RC patients after curative resection.

### Validation of the nomogram

**Fig. 4:**
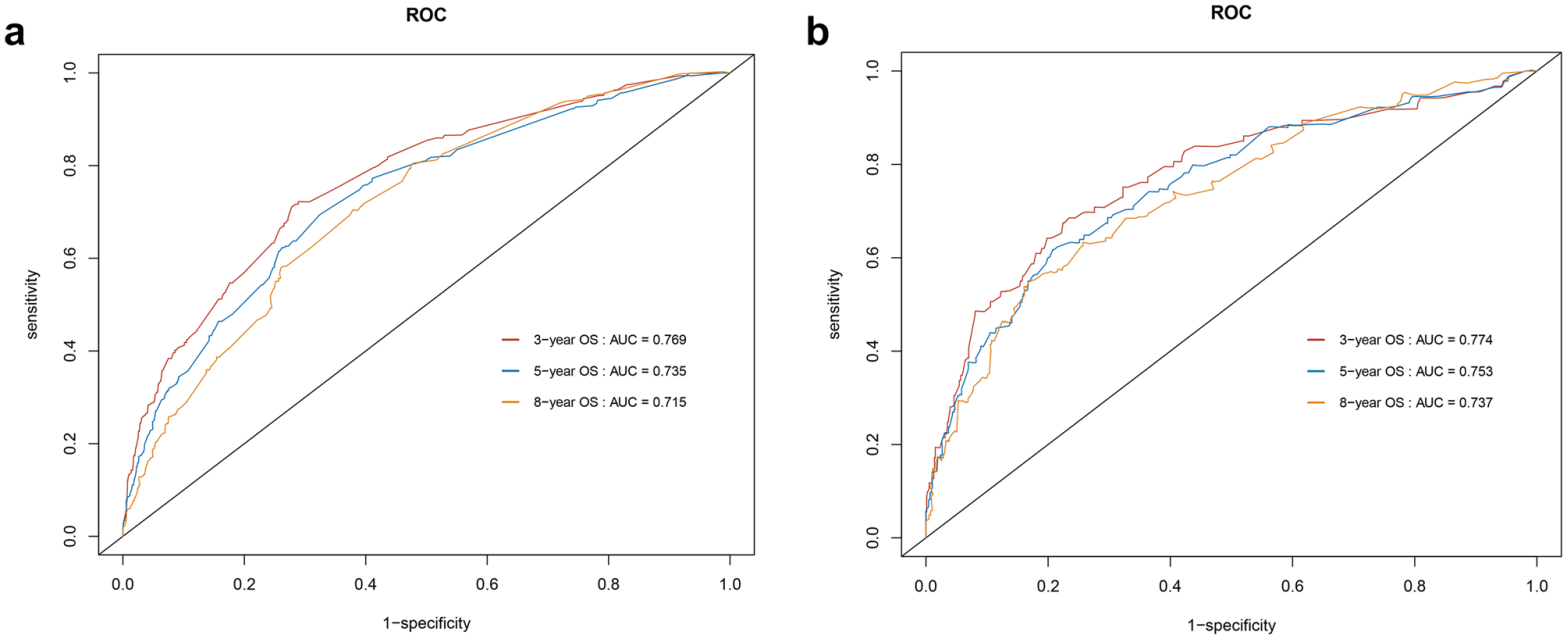
ROC curves for OS prediction of young non-metastatic RC patients after curative resection. **a** 3-, 5-, and 8-year ROC of OS nomogram in training cohort; **b** 3-, 5-, and 8-year ROC of OS nomogram in validation cohort. ROC: receiver operating characteristic; OS: overall survival; RC; rectal cancer

**Fig. 5:**
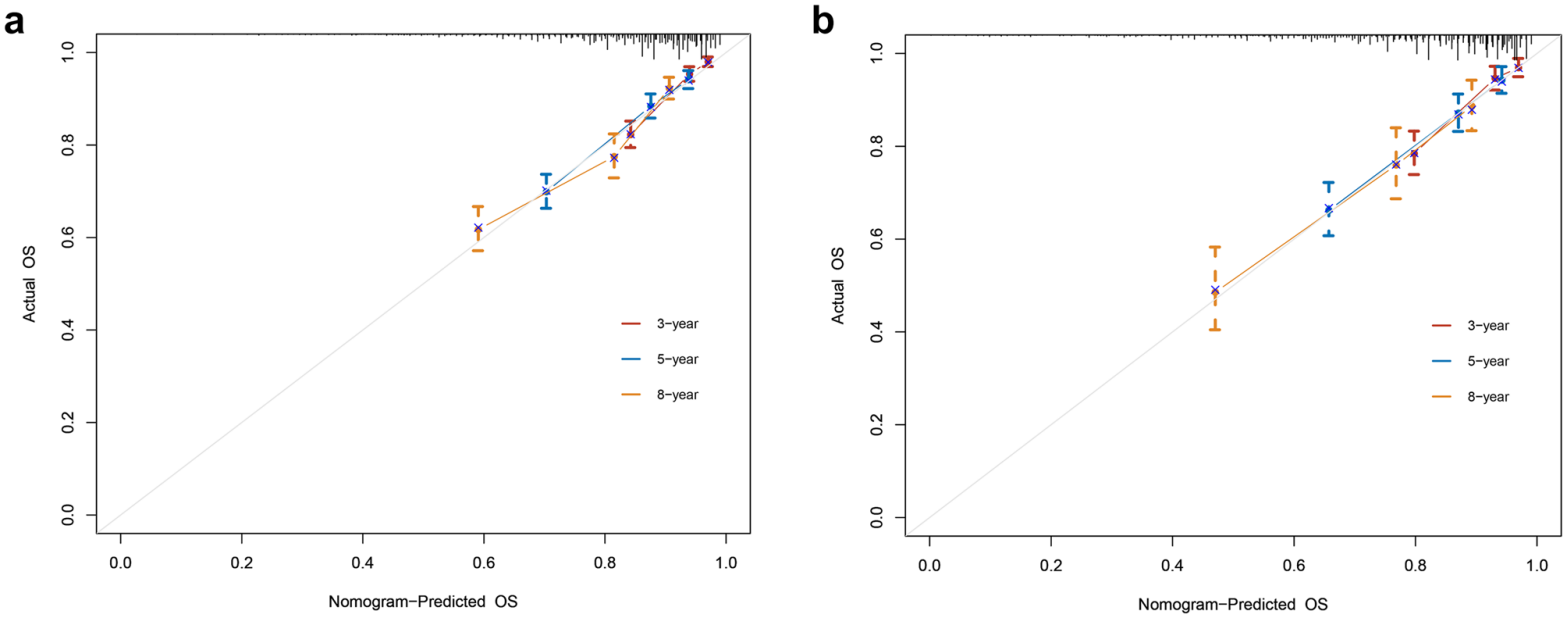
Calibration plots of OS nomogram. **a** The calibration plots of 3-, 5-, and 8-year OS probability in training cohort; **b** the calibration plots of 3-, 5-, and 8-year OS probability in validation cohort. OS: overall survival

**Fig. 6:**
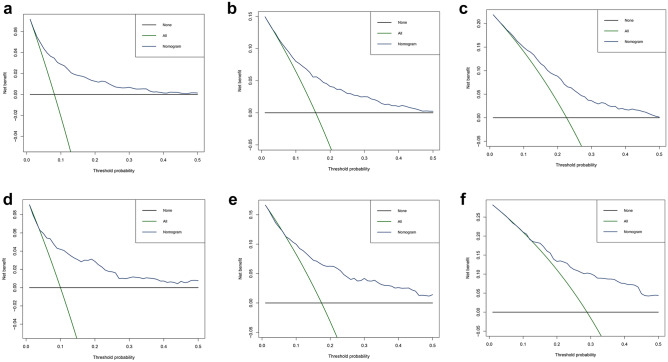
DCA curves of OS nomogram. **a–c** 3-, 5-, and 8-year DCA of OS nomogram in training cohort; **d–f** 3-, 5-, and 8-year DCA of OS nomogram in validation cohort. DCA: decision curve analysis; OS: overall survival

In the present study, C-index, ROC curves, calibration plots, and DCA curves were used to identify the superiority of the nomogram in predicting the prognosis of young non-metastatic RC patients after curative resection. The C-indexes of the nomogram were 0.723 (95% confidence interval (95%CI): 0.709–0.737) in the training cohort and 0.739 (95%CI: 0.719–0.759) in the validation cohort. The C-indexes of the TNM staging system were 0.686 (95%CI: 0.673–0.699) in the training cohort and 0.682 (95%CI: 0.658–0.706) in the validation cohort, both of them were significantly lower than the C-index of the above nomogram (*P* < 0.001). In the ROC curves, a high area under the ROC (AUC) was observed both in the training cohort and validation cohort (Fig. 4). AUC values for 3-, 5-, and 8-year OS of the training cohort were 0.769, 0.735, and 0.715 (Fig. 4a), as for the values of the validation cohort were 0.774, 0.753, and 0.737 (Fig. 4b). Meanwhile, calibration plots presented a good agreement between the actual observation and the nomogram prediction for 3-, 5-, and 8-year OS rates in the training cohort as well as the validation cohort (Fig. 5). Furthermore, DCA results demonstrated that the nomogram model was clinically useful, which can play a practical role in decision-making (Fig. 6).

### Performance of the nomogram in stratification

**Fig. 7:**
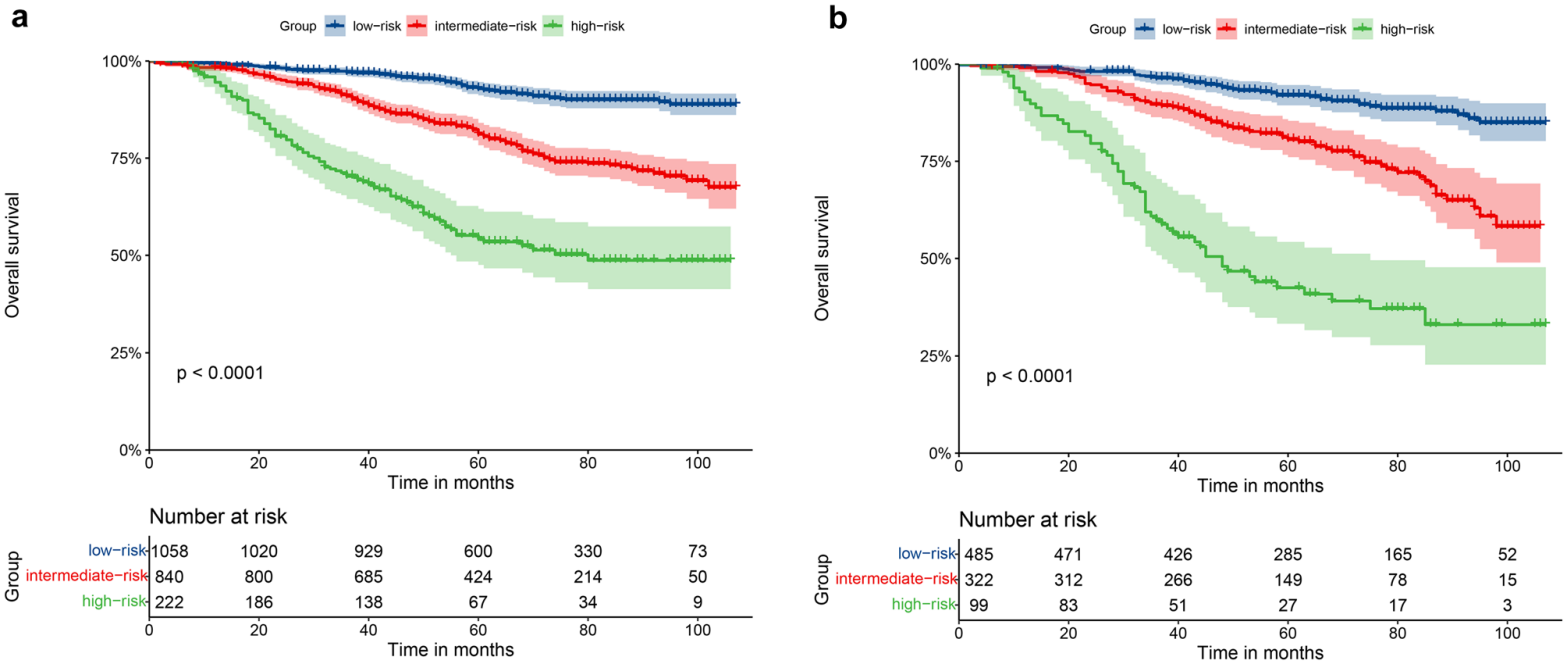
Kaplan–Meier survival curves analysis of OS for young non-metastatic RC patients after surgical resection in the training cohort **a** and validation cohort **b**. OS: overall survival; RC: rectal cancer

All patients were categorized into three subgroups according to the cutoff values of the nomogram for OS: low risk (score ≤ 203), intermediate risk (203 < score ≤ 245), and high risk (245 < score). Kaplan–Meier survival curve analysis indicated that, both in the training cohort and the validation cohort, patients in the high-risk group suffered a significantly poorer prognosis than those in the intermediate-risk group and low-risk group (Fig. 7).

## Discussion

Over the past few years, a dramatic increasing of young RC incidence has been observed in many countries [12–14], which has brought a heavy burden to individuals and the whole society. A study had predicted that the incidence of RC in young patients is expected to increase constantly for decades to come [15]. The cause of this increasing is still not clear. Although some young-onset RC might be due to hereditary cancer syndromes, the majority of patients are sporadic cases [16]. In comparison with elderly patients, young RC patients are more likely to be diagnosed with a later stage of disease [14, 17]. According to statistics, unfavorable histology such as poor differentiation, mucin, and signet ring morphology is also higher in young RC patients [18]. However, survival data for young RC patients are conflicting. Some studies reported a poorer prognosis in young patients [19, 20], while others indicated young patients do not perform worse than elderly individuals [17, 21, 22]. Until now, whether needing the perioperative treatments of young RC patients is mainly guided by the AJCC stage, and the follow-up pattern of all patients after surgery is similar. Unfortunately, it is often found that the disease of some young patients relapse and metastasis after curative resection within a short time. These patients tend to have a shorter overall survival. Also, some low-risk young patients developed irreversible complications after over-treatment, which may seriously affect their quality of life.

In the present study, by using the database of the SEER program, we analyzed data of 3026 young RC patients. All of the analyzed patients were diagnosed with non-metastatic disease and received curative resection. We found that several clinicopathological features including race, histological type, tumor grade, T stage, N stage, CEA level, and number of LN examined were independent prognostic factors for OS. All independent prognosis factors of our study were used to construct an OS nomogram. Except for later tumor and lymph node stage, poor differentiation, mucinous adenocarcinoma, signet ring cell carcinoma, Black race, high preoperative CEA, and examined lymph nodes less than 12 have been proven to be significantly correlated with poor prognosis of young non-metastatic RC patients [23–27]. This study integrated all of the above independent prognosis factors for establishing a nomogram to predict OS in young non-metastatic RC patients after curative resection. A series of validation tests verified the discrimination and reliability of the nomogram model. In a univariate analysis of this study, sex, radiotherapy, chemotherapy, and tumor size were also significantly associated with the prognosis of young RC patients. According to the results of some randomized controlled clinical trials, the present rectal cancer treatment guidelines such as National Comprehensive Cancer Network (NCCN) guidelines recommended that the disease in stage I receives surgical resection alone and the disease in stages II and III receives neoadjuvant chemotherapy with subsequent surgical resection and systemic chemotherapy [28]. Nevertheless, these recommendations are mainly basing on the data of patients over 50 years, with RC patients under 50 years not well studied [29, 30]. A research had reported that for stages II and III disease, young patients seem not to benefit from perioperative treatment [31]. Our results also show that receiving the current chemotherapy and radiotherapy protocols were not the independent good prognostic factors for young non-metastatic RC patients after curative resection. Of course, the effects of chemotherapy and radiotherapy on the prognosis of young RC patients with non-metastatic disease need to be further investigated in more clinical studies. Also, it has been reported that tumor size ≥ 5 cm and male were adverse prognostic factors for patients with RC [32, 33]. However, tumor size and sex failed to be independent prognosis factors in the multivariate analysis of our study, suggesting that these factors may not be critical to OS.

Nomogram is a visual and individualized tool for predicting prognosis. By integrating more clinicopathological variables, nomograms can provide a more accurate prognosis than the TNM staging system [9–11]. At present, several nomograms about rectal cancer had been reported [34–36]. Wang et al. [35] also established a nomogram for predicting cancer-specific mortality in young RC patients with stages I–IV based on the information from the SEER database, and the C-indexes of this model were greater than 0.75, which showed good predictive ability. However, the nomogram neither subdivides T stages and N stages, nor discusses the OS of young RC patients. To our knowledge, the nomogram we constructed is the first one to predict the OS of young non-metastatic RC patients after curative resection. The nomogram presented good predictive ability, with high values of C-indexes (training cohort 0.723 and validation cohort 0.739), which were better than the TNM staging system. Besides that, ROC curves, calibration plots, and DCA curves presented satisfactory performance of the above nomogram. The nomogram was able to identify subgroups of patients at different risks, in which high-risk patients may need intensive therapy and follow-up while low-risk patients should try to avoid over-treatment.

Meanwhile, there remains some limitation. First, since our study is a retrospective design, potential selection bias is hard to eliminate. Second, the SEER database did not contain other important data like genetic mutation state or specific drugs for treatment, which may affect survival outcomes. Last but not least, we only accessed information from the SEER database; it would be better if we get more information from external validation.

## Conclusion

A nomogram was constructed to predict the 3-, 5-, and 8-year OS for young non-metastatic RC patients after curative resection. The nomogram could give exact survival predictions for young non-metastatic RC patients and identify individuals with different prognostic risks for whom an individualized follow-up and treatment plan should be emphasized.
